# Clinical Presentations and Treatment Approaches in a Retrospective Analysis of 128 Intracranial Arteriovenous Malformation Cases

**DOI:** 10.3390/brainsci14111136

**Published:** 2024-11-12

**Authors:** Corneliu Toader, Mugurel Petrinel Radoi, Milena-Monica Ilie, Razvan-Adrian Covache-Busuioc, Vlad Buica, Luca-Andrei Glavan, Christian-Adelin Covlea, Antonio Daniel Corlatescu, Horia-Petre Costin, Carla Crivoi, Leon Danaila

**Affiliations:** 1 Department of Neurosurgery, “Carol Davila” University of Medicine and Pharmacy, 020021 Bucharest, Romania; corneliu.toader@umfcd.ro (C.T.); milena-monica.ilie0720@stud.umfcd.ro (M.-M.I.); razvan-adrian.covache-busuioc0720@stud.umfcd.ro (R.-A.C.-B.); vlad.buica0720@stud.umfcd.ro (V.B.); luca-andrei.glavan0720@stud.umfcd.ro (L.-A.G.); christian-adelin.covlea0720@stud.umfcd.ro (C.-A.C.); antonio.corlatescu0920@stud.umfcd.ro (A.D.C.); horia-petre.costin0720@stud.umfcd.ro (H.-P.C.); acad.leondanaila@gmail.com (L.D.); 2 Department of Vascular Neurosurgery, National Institute of Neurology and Neurovascular Diseases, 077160 Bucharest, Romania; 3 Faculty of Mathematics and Computer Science, University of Bucharest, 010014 Bucharest, Romania; crivoicarla02@gmail.com; 4 Romanian Academy, Medical Sciences Section, 010071 Bucharest, Romania

**Keywords:** arteriovenous malformation, Spetzler–Martin grading system, surgery, radiosurgery, endovascular embolization

## Abstract

Background: Intracranial AVMs are a highly heterogeneous group of lesions that, while not very common, can pose significant risks. The therapeutic management of AVMs is complicated by ambiguous guidelines, particularly regarding which Spetzler–Martin grades should dictate specific treatment options. This study analyzed the clinical presentations and treatment approaches of 128 brain AVM cases managed between 2014 and 2022 at the National Institute of Neurology and Neurovascular Diseases in Bucharest, Romania. Methods: A retrospective analysis was conducted on patient demographics, clinical symptoms, Spetzler–Martin categorization, nidus localization, therapeutic management, and outcomes. Statistical analysis was performed using Python 3.10. Results: In our cohort of patients, the median age was 45 years, with a slight male predominance (67 males, 61 females). At admission, 51.5% presented with elevated blood pressure. The majority of patients had a Spetzler–Martin score of 2 (37.5%), followed by scores of 3 (31.3%) and 1 (20.3%). Treatment strategies included microsurgical resection in 32% of cases, conservative management in 31.2%, Gamma Knife radiosurgery in 22.6%, and endovascular embolization in 13.3%. Notably, open surgery was predominantly chosen for Grade II AVMs. The functional outcomes were favorable, with 69.5% achieving a good recovery score on the Glasgow Outcome Scale. Only four in-hospital deaths occurred, all in patients who underwent open surgery, and no deaths were recorded during the two-year follow-up. Conclusions: AVMs within the same Spetzler–Martin grade display considerable complexity, necessitating personalized treatment strategies. Our findings highlight the limitations of open surgery for Grade I cases but affirm its effectiveness for Grade II AVMs.

## 1. Introduction

Intracranial arteriovenous malformations (AVMs) are congenital cerebrovascular anomalies characterized by direct, high-pressure connections between arteries and veins without intermediary capillaries, forming a nidus of dysplastic vessels [1]. This structural deficiency allows blood to flow directly from arteries to veins, leading to vessel dilation and tortuosity, significantly increasing the risk of hemorrhage and neurological impairment [2]. Although AVMs can occur throughout the body, intracranial AVMs are particularly concerning due to their heightened bleeding risk.

The true prevalence of brain AVMs (bAVMs) remains uncertain, largely because many cases are asymptomatic. Postmortem analyses suggest a prevalence between 5 and 613 cases per 100,000, while epidemiological studies estimate an incidence of 1.12 to 1.42 per 100,000, with hemorrhage as the first presenting symptom in 38–68% of cases [3,4]. Advances in MRI technology have increased the detection of unruptured AVMs, while the incidence of ruptured cases remains stable [2]. Most symptomatic patients are diagnosed between the ages of 20 and 50, with no significant gender differences in prevalence [2,5]. There is not a significant difference in incidence between males and females [1]. Hemorrhage occurs in approximately 65% of cases, most commonly in parenchymal regions (82%), followed by intraventricular and subarachnoid sites [5,6,7]. Risk factors for rupture include frontal lobe location, deep venous drainage, deep nidus location, and associated aneurysms [8,9,10,11]. Pregnancy as a risk factor is debated, with mixed evidence on increased rupture risk [5,12,13]. Other common symptoms include seizures, headaches, and neurological deficits [5].

The etiology of bAVMs is poorly understood, though likely congenital. Deficient capillary formation during fetal development may play a role [2]. Syndromic associations, such as hereditary hemorrhagic telangiectasia (Rendu–Weber–Osler syndrome), Cobb syndrome, and cerebrofacial arteriovenous metameric syndromes, suggest a genetic component in some cases [4,14,15]. Abnormal angiogenesis, vasculogenesis, and inflammation, mediated by factors such as VEGF, angiopoietin-2, TGF-β, and MMPs, are implicated in AVM development [10,16,17,18].

Grading systems assess AVM morbidity and mortality risk, with the Spetzler–Martin scale being a primary tool, evaluating nidus size, location eloquence, and venous drainage type. Eloquent areas include sensorimotor, language, and visual cortices, as well as deep brain structures like the hypothalamus, thalamus, and brainstem. The Lawton–Young scale and Spetzler–Ponce classification further refine risk and outcome prediction [4,10].

Treatment strategies for bAVMs include microsurgical resection, endovascular embolization, and stereotactic radiosurgery, especially Gamma Knife radiosurgery. When surgical risks are high, conservative management focuses on symptom control and hemorrhage prevention through antiepileptic drugs (AEDs), monitoring, and lifestyle adjustments [19]. For inoperable AVMs, alternative therapies such as Botox injections for refractory migraine management have shown symptom relief [20].

The management of unruptured AVMs remains debated. The ARUBA trial (2014) suggested medical management as preferable for unruptured AVMs over surgical interventions, though critiques highlight limitations such as early termination, selection bias, and broad inclusion criteria [11,21,22]. Despite these limitations, the ARUBA trial has influenced a trend toward conservative management in unruptured cases [23].

While significant data on AVMs exist globally, particularly from high-resource healthcare settings, regional data from Romania remain scarce due to limited national statistics and centralized reporting. This study offers novel insights into AVM cases in Romania by providing a comprehensive assessment of patient demographics, clinical presentations, and treatment strategies based on nidus location and Spetzler–Martin grading. Given the constraints and unique aspects of the Romanian healthcare system, our findings may inform localized treatment protocols, guide healthcare policy, and support the better allocation of resources for multidisciplinary AVM management [24].

## 2. Materials and Methods

This study presents a retrospective unicentric analysis of 128 cases of cerebral AVMs treated at the Department of Neurosurgery, National Institute of Neurology and Neurovascular Diseases in Bucharest, Romania, between 2014 and 2022. Patients were subjected to one of four management approaches: microsurgical resection, Gamma Knife radiosurgery, endovascular embolization, or conservative treatment as determined by the medical team. A comprehensive analysis was conducted, focusing on specific variables throughout the preoperative, intraoperative, and postoperative stages of patient medical management. Particular attention was given to factors such as bleeding, presence of arterial hypertension, seizures, location of the nidus, Spetzler–Martin score, and hemorrhage. Additionally, postoperative complications were discussed, and the number of reoperations was noted.

The research adhered to the main principles of the Declaration of Helsinki and received approval from the Ethics Committee of the National Institute of Neurology and Neurovascular Diseases in Bucharest, Romania (Approval No. [7230]). Clinical data, including age, sex, Glasgow Coma Scale score, bleeding, and treatment, were extracted from relevant patient files. All data processing was conducted in compliance with the current General Data Protection Regulation (GDPR), and informed consent was obtained from all patients included in this study.

Statistical analysis and figure plotting were performed using Python version 3.10, developed by the Python Software Foundation (9450 SW Gemini Dr., ECM# 90772, Beaverton, OR 97008, USA). The analysis utilized Python libraries such as pandas, numpy, seaborn, and matplotlib.

## 3. Results

### 3.1. Patient Demographics and Comorbidities

#### 3.1.1. Age and Sex

**Figure 1:**
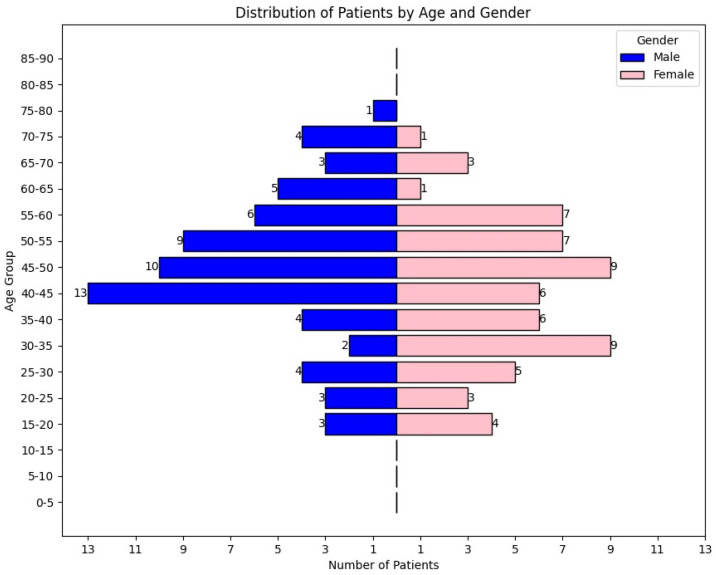
Distribution of patients with intracranial AVM by age and gender.

A dataset comprising 128 intracranial AVM cases treated between 2010 and 2022 was collected from the National Institute of Neurology and Neurovascular Diseases in Romania. We analyzed (Figure 1) the demographic characteristics of patients diagnosed with AVMs, focusing on age and gender distribution. Among the patients, 67 were male and 61 were female. The median age at diagnosis was 45 years, and the mean age was 44.5 years. The highest prevalence was observed in the 40–50 age group, which accounted for 29.6% of the total cases.

#### 3.1.2. Arterial Hypertension at Admission Time

**Figure 2:**
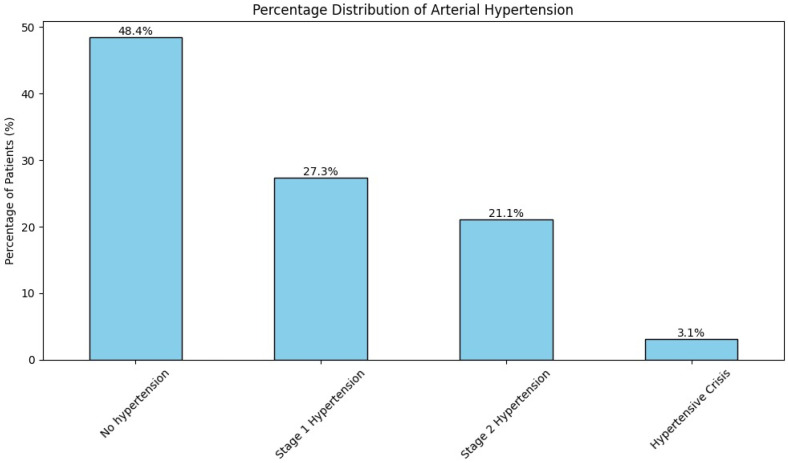
Assessment of arterial hypertension of intracranial AVM patients.

Arterial blood pressure was measured at the time of admission for all patients. Of these, 51.5% (n = 66) had systolic and diastolic pressures exceeding 140/90 mmHg. Specifically, 27.3% (n = 35) had stage 1 hypertension, 21.1% (n = 27) had stage 2 hypertension, and 3.1% (n = 4) experienced a hypertensive crisis (Figure 2).

### 3.2. Presentation

#### 3.2.1. Glasgow Coma Scores

**Figure 3:**
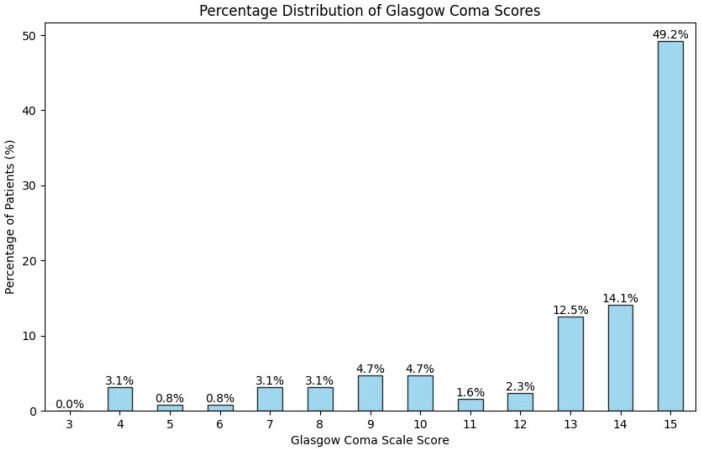
Distribution of patients by Glasgow Coma Scale.

At presentation, the mental state of the patients was assessed using the Glasgow Coma Scale. The majority of patients, 75.8% (n = 97), had minor brain injuries with scores of 13 or higher. Specifically, 49.2% (n = 63) had a score of 15, 14.1% (n = 18) had a score of 14, and 12.5% (n = 16) had a score of 13 (Figure 3).

#### 3.2.2. Headaches, Nausea and Vomiting

A total of 51.6% (n = 66) of patients presented with headaches, characterized by persistent and severe headaches, while 32.8% (n = 42) experienced episodes of vomiting prior to admission.

#### 3.2.3. Comitial Seizures

**Figure 4:**
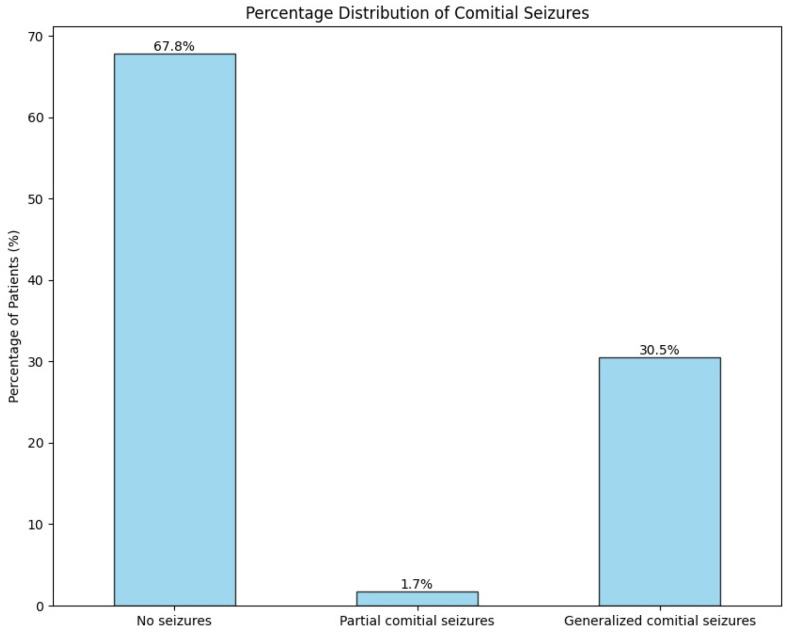
Percentage distribution of comitial seizures in intracranial AVM patients.

A history of epilepsy was present in 32.2% (n = 41) of the patients, either under focal or generalized seizures (Figure 4).

#### 3.2.4. Spetzler–Martin Scores

**Figure 5:**
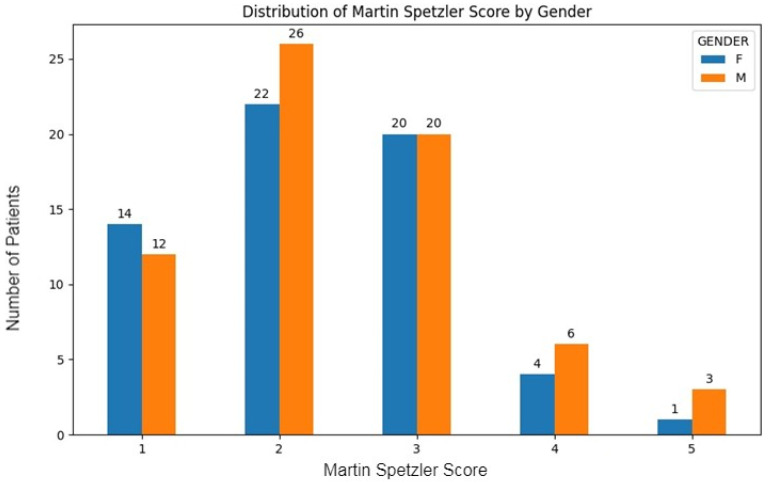
Distribution of Spetzler–Martin score by gender in intracranial AVM patients.

Among the patients, 20.3% (n = 26) had a score of 1, indicating a nidus smaller than 3 cm, a non-eloquent location, and venous drainage into the superficial system. The majority of patients had a score of 2, accounting for 37.5% (n = 48), while 31.3% (n = 40) had a score of 3. Scores of 4 and 5 were less common, with 7.8% (n = 10) and 3.1% (n = 4) of patients, respectively (Figure 5).

### 3.3. Rupture Status Resulting in Intracranial Hemorrhage

**Figure 6:**
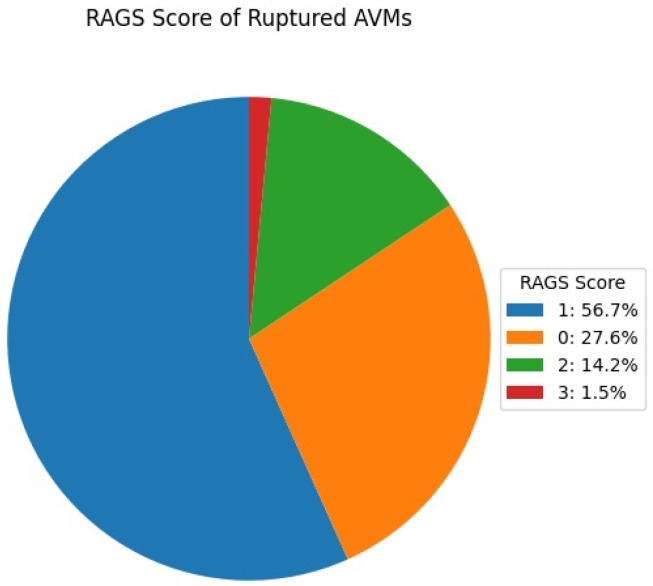
Percentage distribution of patients with intracranial AVM by RAGS.

In this patient cohort, 63.3% (n = 81) of patients presented with unruptured AVMs, while 36.7% (n = 47) of patients had ruptured AVMs, resulting in intracranial hemorrhage. Of those with ruptures, 20.3% (n = 26) of patients experienced subarachnoid hemorrhage. The ruptured cases were assessed according to the RAGS classification (Figure 6), with distributions as follows: 56.7% of ruptured AVMs were scored as RAGS 1, indicating minimal rupture risk, followed by 27.6% as RAGS 2, 14.2% as RAGS 3, and 3.5% as RAGS 4. This grading reflects the extent and severity of hemorrhagic events, aiding in clinical stratification and management decisions for patients with ruptured AVMs.

### 3.4. Cases Management

**Figure 7:**
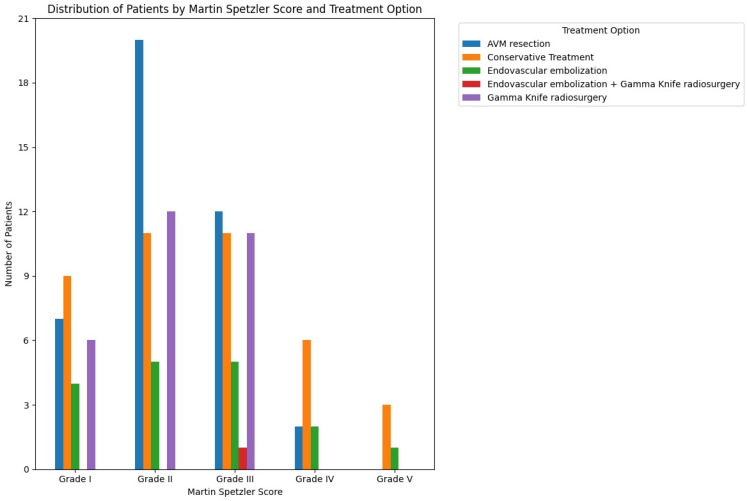
Distribution of patients with intracranial AVM by treatment and Spetzler–Martin score.

**Figure 8:**
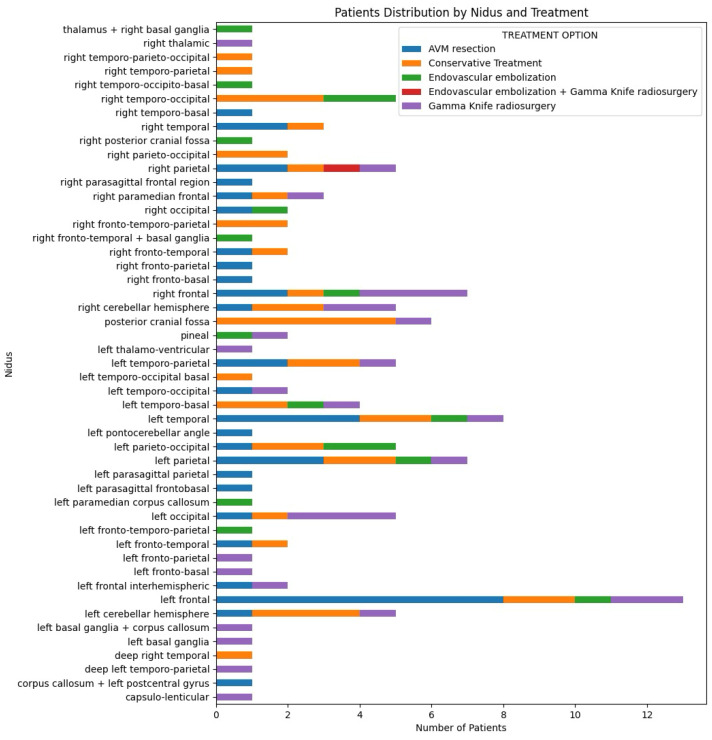
Distribution of nidus locations and treatment options in AVM patients.

This study identified five distinct treatment options for patients with intracranial AVMs, distributed according to the Spetzler–Martin score (Figure 7) and the nidus localization (Figure 8). Microsurgical AVM resection was chosen for 32% of patients (n = 41), while conservative treatment was selected for 31.2% (n = 40). Gamma Knife radiosurgery was used for 22.6% (n = 29), and endovascular embolization was applied to 13.3% (n = 17). A combination of endovascular embolization and Gamma Knife radiosurgery was used for 0.8% (n = 1).

Figure 7 illustrates a marked preference for open surgery in the treatment of Grade II intracranial AVMs.

### 3.5. Short-Term Outcomes

**Figure 9:**
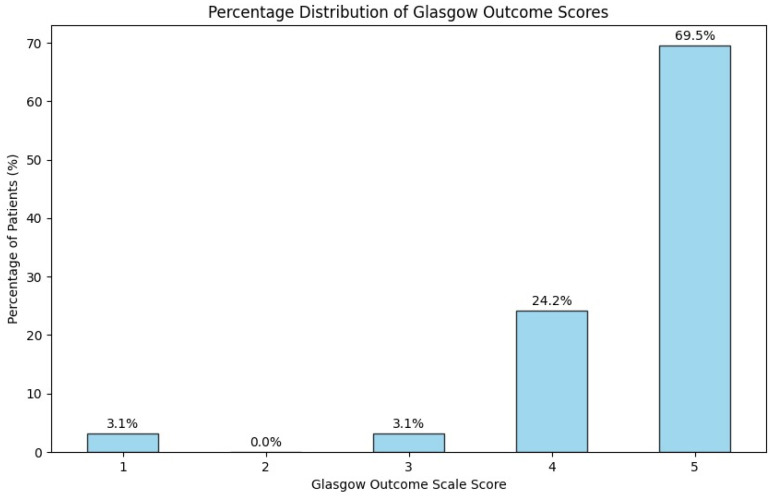
Distribution of Glasgow Outcome Scores in patients with intracranial AVM.

Functional outcomes were assessed before hospital discharge. According to the Glasgow Outcome Scale (Figure 9), the majority of patients (69.5%, n = 89) achieved a score of 5, indicating good recovery. A smaller percentage, 24.2% (n = 31), had a score of 4, reflecting moderate disability. Scores of 1 and 3 were observed in 3.1% (n = 4) of patients each, indicating death or persistent disabilities. No patients received a score of 2. Only four in-hospital deaths were reported, all occurring in patients who underwent open surgery. All patients were followed-up for a minimum of two years, with some patients receiving extended follow-up based on clinical need and availability.

This approach ensured consistent post-treatment monitoring and allowed for the comprehensive assessment of long-term outcomes across the cohort.

## 4. Discussion

The literature (Table 1) exposes a diverse range of findings regarding AVM treatment outcomes across various patient populations. Studies have examined numerous facets of AVM management, including patient demographics, Spetzler–Martin grading, treatment modalities, and key clinical outcomes. Collectively, these studies shed light on important patterns and challenges in AVM treatment, offering valuable insights for contemporary clinical practice.

Although the exact mechanism by which epilepsy occurs in patients with AVMs is not fully understood, several risk factors have been identified that increase the likelihood of seizures. These include younger age, temporal lobe location, cortical involvement, and a nidus diameter exceeding 3 cm [34]. In our cohort, we observed that out of 41 patients with AVMs located in or involving the temporal region, 17 experienced epileptic seizures. These seizures were either focal or progressed to generalized seizures, supporting the association between temporal lobe AVMs and increased seizure incidence. All patients experiencing seizures were prescribed oral AEDs at discharge to maintain effective seizure control and manage their condition in the long term.

The ARUBA trial did not evaluate surgical outcomes for patients with Grade I or II AVMs, who are considered optimal candidates for surgery [35]. This limitation reduces the trial’s ability to provide comprehensive recommendations. Therefore, we aim to address treatment option controversies and present our own findings to offer a more inclusive perspective.

In contrast to the ARUBA trial findings, a study by Potts et al. concluded that surgery remains the “gold standard” treatment for most low-grade AVMs. This study emphasized using endovascular embolization as a preoperative adjunct. High surgical cure rates and excellent functional outcomes in patients with both ruptured and unruptured AVMs support a strong preference for surgical intervention, offering the best cure rate, lowest risk profile, and greatest protection against hemorrhage for low-grade AVMs [36]. A prospective study by Baharvahdat et al. demonstrated that endovascular treatment (EVT) was highly effective for low-grade AVMs classified as Spetzler–Martin I–II. This study reported a high rate of complete exclusion with a low complication rate of 5%. EVT was recommended as the first-line treatment for both ruptured and unruptured low-grade AVMs located in deep or eloquent regions, where the risks of open surgery are significant [37]. Out of 48 patients with Spetzler–Martin Grade II AVMs, 20 (41.6%) underwent microsurgical resection, supporting Potts’s assertion that open surgery is the gold standard for low-grade AVMs. Conversely, among 26 Grade I AVMs, 9 received conservative treatment and only 7 underwent microsurgical resection, indicating a decline in the preference for open surgery in these cases and supporting ARUBA’s claims. Our findings suggest that while open surgery may be the gold standard for Grade II AVMs, it is not necessarily the optimal solution for all Grade I AVMs.

In line with Lawton’s proposal to further classify Grade III AVMs into subtypes such as S1V1E1, S2V1E0, S2V0E1, and S3V0E0, our cohort also reflects the heterogeneous nature of these AVMs [38]. The data show no clear preference for treatment options among patients with Grade III AVMs, as similar numbers underwent AVM resection, Gamma Knife therapy, and conservative treatment. This diversity in treatment approaches underscores the complexity and variability within Grade III AVMs, supporting the idea of further subclassification to better tailor treatment strategies [38]. Notably, conservative treatment is typically recommended for high-grade AVMs, highlighting the nuanced decision-making required for Grade III cases.

Stereotactic radiosurgery (SRS) achieves a 70–80% obliteration rate for bAVMs. Recognizing that the effects of SRS are delayed, the annual hemorrhage rate during the latency period between radiation and complete nidus obliteration following Gamma Knife radiosurgery (GKRS) was found to be 1.4%, which is lower than the general rate of 2–4% [32,39]. In our cohort, GKRS was selected exclusively for Grade I to III AVMs when open surgery was unequivocally declined due to patient comorbidities, patient refusal, or when the AVM was located in a deep area that made surgical access challenging (such as basal ganglia or corpus callosum). The case of the single patient who underwent EVT followed by SRS further supports the recommendations for multimodal approaches in managing Grade III and IV AVMs [35,40]. Aside from Grade V AVMs in older patients, there is no established consensus on conservative management, particularly in light of critiques following the ARUBA study [35]. In our patient cohort, the use of medications such as AEDs, analgesics, and antihypertensive drugs for hemorrhagic stroke control, combined with regular monitoring during follow-ups, resulted in no deaths over the strict two-year follow-up period. This outcome was observed in patients who received only conservative management, meaning that they did not undergo any of the aforementioned procedures.

## 5. Conclusions

Our study reveals previously unrecognized distinctions within Spetzler–Martin Grade II AVMs, uncovering that nuanced patient and AVM characteristics—such as lesion depth, vascular complexity, and the presence of hypertension—significantly direct optimal treatment pathways. Contrary to generalized protocols, our data suggest that microsurgery provides superior outcomes for Grade II AVMs in eloquent regions, whereas GKRS proves particularly effective for deep-seated AVMs in hypertensive patients, where traditional risk models might discourage intervention. Furthermore, we identified that EVT is most beneficial in non-eloquent, less complex vascular structures, demonstrating that tailored treatment approaches guided by these newly elucidated factors can substantially improve patient outcomes. These findings challenge existing paradigms, advocating for a refined treatment framework that integrates detailed anatomical and comorbidity profiles to enhance therapeutic precision and reduce risks associated with AVM management.

## Figures and Tables

**Table 1:** Literature review table that analyzes significant studies about AVM and treatment outcomes.

Study	Population (n)	Male-to-Female Ratio	Spetzler–Martin Grades Included	Variables Assessed, Similarly to Our Study	Treatment Method	Key Findings
Von Der Brelie et al. [25]	293 (out of which 103 presented with epilepsy and had follow-up)	59 males/44 females	I–III	Seizure outcomes, hemorrhage, treatment type	Microsurgery	Favorable seizure outcomes post-surgery; seizure control improved in AVM patients post-resection
De Castro-Afonso et al. [26]	203 (117 unruptured AVMs—86 ruptured AVMs)	108 males/95 females	I–IV	Venous drainage, nidus size, AVM rupture	Microsurgery, EVT	Larger draining veins linked to higher hemorrhage risk; supports aggressive treatment for high-risk AVMs
Nesvick et al. [27]	352	150 males/202 females	I–III	Obliteration rates, biological effective dose (BED)	Stereotactic radiosurgery	BED > 133 Gy predicts high obliteration rates post-SRS; recommended dose adjustments for AVM obliteration
Steiner et al. [28]	247	132 males/115 females	I–IV	Treatment outcomes, angiographic obliteration	Gamma Knife radiosurgery	High obliteration rates with SRS; few adverse events, supporting radiosurgery for specific AVM grades
Heros et al. [29]	153	83 males/70 females	I–V	Surgical outcomes, hemorrhage, neurological deficits	Surgical resection	Surgical resection beneficial for Grades I-III, less so for IV-V; conservative management recommended for higher grades
Redekop et al. [30]	97 patients with AVM and intranidal aneurysm	52 males/45 females	I–III	Aneurysm presence, hemorrhage risk	EVT, microsurgery	Intranidal aneurysms increase hemorrhage risk; surgical intervention recommended for associated aneurysms
Mast et al. [31]	281	133 males/148 females	I–IV	Initial hemorrhage, re-bleed risk	EVT, resection, SRS	Initial hemorrhage predicts higher re-bleed risk; suggests monitoring high-risk AVMs post-initial bleed
Al Shahi et al. [32]	92	49 males/43 females	I–IV	Detection rates, public health impact	Not specified	Provides baseline AVM detection rates; calls for resource allocation for AVM management
Maruyama et al. [33]	500	287 males/213 females	I–IV	Hemorrhage risk post-SRS	Gamma Knife radiosurgery	SRS reduces hemorrhage risk; highlights latency period where hemorrhage risk remains until obliteration

## Data Availability

The data that support the findings of this study are available from the corresponding author upon reasonable request. The data are not publicly available due to privacy and ethical restrictions.
